# Expression and significance of the TLR4/MyD88 signaling pathway in ovarian epithelial cancers

**DOI:** 10.1186/1477-7819-10-193

**Published:** 2012-09-17

**Authors:** Ki Hyung Kim, Moo Sung Jo, Dong Soo Suh, Man Soo Yoon, Dong Hun Shin, Jeong Hee Lee, Kyung Un Choi

**Affiliations:** 1 Department of Obstetrics and Gynecology, School of Medicine, Pusan National University, Beomeo-ri, Mulgeum-eup, Yangsan-si, Gyeongsangnam-do, 626-770, Republic of Korea; 2 Biomedical Research Institute and Pusan Cancer Center, Pusan National University Hospital, Busan, Korea; 3 Department of Medicine School of Medicine, Pusan National University, Beomeo-ri, Mulgeum-eup, Yangsan-si, Gyeongsangnam-do, 626-770, Republic of Korea; 4 Department of Pathology, Pusan National University Yangsan Hospital, Beomeo-ri, Mulgeum-eup, Yangsan-si, Gyeongsangnam-do, 626-770, Republic of Korea; 5 Department of Pathology, School of Medicine, Pusan National University, Beomeo-ri, Mulgeum-eup, Yangsan-si, Gyeongsangnam-do, 626-770, Republic of Korea

**Keywords:** Toll-like receptor, MyD88, Ovarian epithelial cancer

## Abstract

**Background:**

Toll-like receptors (TLR) are a family of pattern recognition receptors that constitutes a major part of the innate immune system. The TLR4/(Myeloid differentiation factor 88 (MyD88) signaling pathway has been shown to have oncogenic effects.

**Methods:**

To demonstrate the role of TLR4/MyD88 signaling in ovarian epithelial cancers (OECs), we examined the expression of TLR4, MyD88 and nuclear factor- κB (NF-κB) in OECs. The expression of TLR4, MyD88, and NF-κB was detected by immunohistochemistry, and the relationships between these and clinicopathologic features in 123 cases of OECs were also analyzed.

**Results:**

The expression of TLR4, MyD88, and NF-κB in OECs was observed in 46.3% (57/123), 36.6% (45/123) and 65% (80/123) of OEC cases, respectively. The TLR4, MyD88, and NF-κB expressions were associated with the histologic type of OECs, particularly with the clear cell type of OEC. There was no significant correlation between TLR4 or NF-κB expression and histologic grade, tumor size, mitotic count, FIGO (International Federation of Gynecology and Obstetrics) stage, disease recurrence. However, there was a significant correlation between MyD88 expression and FIGO stage, disease recurrence as well as histologic type. In univariate analysis, the expression of TLR4 and MyD88, and the coexpression of TLR4/MyD88 and TLR4/MyD88/NF-κB had a significant impact on the survival of patients with OECs. Only MyD88 expression had an independent prognostic significance in multivariate analysis.

**Conclusions:**

Our findings suggest that the TLR4/MyD88 signaling pathway is associated with the survival of patients with OECs, and that MyD88 is an independent prognostic predictor in patients with OECs. The TLR4/MyD88 signaling pathway may be a mechanism responsible for poor prognosis in patients with clear cell type of OEC.

## Background

Ovarian epithelial cancer (OEC) is the second most common and the most lethal of all gynecologic malignancies. Early detection of OEC is difficult because there is no specific screening tool and long-term survival has not been significantly prolonged although many advances have been made in the treatment of OEC. Many investigators have tried to understand the biology of OEC and identify the mechanisms of chemoresistance, which is one of the major causes of treatment failure for OEC.

The toll-like receptors (TLRs) are surface molecules on eukaryotic cells that detect and respond to microbial infection. TLRs are the best studied of a class of host receptors known as pattern recognition receptors (PRRs). TLRs are central to the regulation of host protective adaptive immune responses. In humans, 13 types of TLRs have been identified, and are mainly expressed by immune cells and epithelial cells. Recently, TLRs have also been detected in many tumor cell lines or tumors, especially epithelial-derived cancers [1]. Recent evidence has shown that functional TLRs are expressed on a wide variety of tumors [2]. TLRs may promote the proliferation and inhibit the apoptosis, and lead to migration, invasion and angiogenesis of tumors [3]. Most TLRs share a common adaptor molecule, myeloid differentiation primary-response protein (MyD88), to activate nuclear factor- κB (NF-κB) and mitogen-activated protein kinases (MAP kinases) and induce expression of various inflammatory cytokine genes [4]. In a murine model of liver carcinogenesis induced by injection of the mutagen diethylnitroseamine, MyD88-dependent signaling has been shown to be critical for tumorigenesis [5]. MyD88 has also recently been shown to be crucial for tumor promotion in models of spontaneous and carcinogen-induced (azoxymethane) intestinal tumorigenesis [6,7].

TLRs are supposed to be expressed in the female genital tract and may play an essential role in the defense against microbes [8]. Current studies suggest a link between TLR signaling and tumorigenesis and tumor progression in the human female reproductive tract. Recent work has also suggested the role of TLR4 in the propagation of OECs [9]. They showed that TLR4 and MyD88 were expressed in tumor cells of OEC both at the mRNA and protein level, and that the TLR4 induces NF-κB activation in MyD88-positive OEC cells. They also suggested that the MyD88 status of OCE cells determines the apoptotic response to paclitaxel. However, the data available so far are still limited and most evidences are the results of *in vitro* research on cell lines. Also, the biological significances of TLRs expressed on tumor cells of the genital tract have not been fully elucidated yet.

We propose that the TLR4/MyD88 signaling pathway may be a risk factor for developing cancer and may represent a novel target for the development of biomodulators for treatment of OECs. We tried to determine the expression and prognostic associations of TLR4/MyD88 pathway proteins in various histologic types of OECs. In this study, we performed an immunohistochemical analysis of TLR4, MyD88 and NF-κB expression and analyzed the associations between their immunoreactivity and clinicopathologic features in OECs.

## Methods

### Patients and tissue

A total of 123 patients with OECs who underwent surgery and were diagnosed between 1998 and 2008 were selected from the archives of the Pusan National University Hospital for this study. All the cases were of primary OECs. Hematoxylin and eosin (H&E) stained sections were reviewed and subclassified according to the WHO (World Health Organization) guidelines. The tumor stage was determined by FIGO (International Federation of Gynecology and Obstetrics) classification, and cases was classified by the Silverberg tumor grade system. The mean age of patients at the time of surgery was 51 years (range, 15 to 82 years). Clinicopathologic characteristics are shown in Table 1. All patients, except those with grade 1, stage IA, were given adjuvant chemotherapy consisting of platinum/Taxol-containing agents. They were followed up for 3 to 140 months (mean follow-up period, 43 months). During this follow-up period, 61 patients (49.6%) developed recurrent disease and 31 patients (25.2%) died from the disease. Normal ovaries obtained from ten patients who underwent surgery for benign gynecologic disease were included as a control. Written informed consent was obtained from the patient for publication of this report and any accompanying images. Ethical approval for the project was obtained from the Pusan National University Hospital Research Ethics Committee (PNUH IRB #2010085).

**Table 1:** Patient and tumor characteristics

**Characteristics**		**N (%)**
Histologic type	Serous	60 (48.8)
	Mucinous	25 (20.3)
	Endometrioid	11 (8.9)
	Clear cell	25 (20.3)
	Undifferentiated	2 (1.6)
Histologic grade	Grade 1	35 (28.5)
	Grade 2	62 (50.4)
	Grade 3	26 (21.1)
Tumor size	<10 cm	61 (49.6)
	≥10 cm	62 (50.4)
Mitotic count	<10/10HPFs	50 (40.6)
	10 ≤ but < 20/10HPFs	44 (35.8)
	≥20/10HPFs	29 (23.6)
FIGO stage	I	54 (43.9)
	II	8 (6.5)
	III	44 (35.8)
	IV	17 (13.8)
Disease recurrence	No	62 (50.4)
	Yes	61 (49.6)
Survival	Alive	92 (74.8)
	DOD	31 (25.2)

### Immunohistochemistry

The tissue specimens were fixed in 10% formalin and embedded in paraffin. Slides, 4 μm in thickness, were deparaffinized in xylene and rehydrated through a series of graded ethanol. Endogenous peroxidase activity was blocked by incubation with 3% hydrogen peroxide in methanol for 10 minutes. Antigen retrieval was performed by microwaving the slides in citrate buffer (pH 6.0). The sections were then incubated at 4°C overnight with anti-TLR4 antibody (mouse monoclonal, HTA125, 1:100; eBioscience, San Diego, CA, USA), anti-MyD88 antibody (rabbit polyclonal, HFL-296, 1:100; Santa Cruz Biotech, Santa Cruz, CA, USA), or anti-NF-κB p65 antibody (mouse monoclonal, F-6, 1:100; Santa Cruz Biotech, Santa Cruz, CA, USA). Immunoreactivity was visualized using 3,3'-diaminobenzidine (DAB, DakoCytomation, Glostrup, Denmark). Slides were counterstained with Meyer's hematoxylin. Human tonsil tissue was used as a positive control and phosphate-buffered saline without the primary antibody served as a negative control.

Each slide was evaluated independently by two pathologists who were blinded to clinical and outcome data. The assessment of TLR4, MyD88, and NF-κB expression was based on previously described guidelines [10,11]. Their expressions were assessed semi-quantitatively based on the percentage of stained tumor cells and the staining intensity. The percentage of positive tumor cells was rated as follows: 1 point, ≤ 10% positive tumor cells; 2 points, 11%-50% positive tumor cells; 3 points, 51%-80% positive tumor cells; and 4 points, ≥81% positive tumor cells. The staining intensity was rated as follows: 1 point, weak intensity; 2 points, moderate intensity; and 3 points, strong intensity. Points for expression and percentage of positive cells were added, and specimens were attributed to four groups according to their overall score: negative, ≤10% of cells stained positive, regardless of intensity; weak expression, 3 points; moderate expression, 4 to 5 points; and strong expression, 6 to 7 points. Weak expression was rated as negative, and moderate and strong expressions were rated as positive for analysis.

### Statistical analysis

For statistical analysis, SPSS 15.0 (SPSS Inc. Chicago, IL, USA) was used. The *χ*^2^ test was used to evaluate the correlation between the expression of TLR4, MyD88, and NFκB, and the clinicopathologic parameters. The linear correlations for expression were assessed using the Pearson correlation coefficient. Survival analysis was performed using the Kaplan-Meier method; univariate and multivariate Cox regression analyses were used to identify variables associated with overall survival (OS). *P* values of < 0.05 were considered statistically significant.

## Results

**Figure 1:**
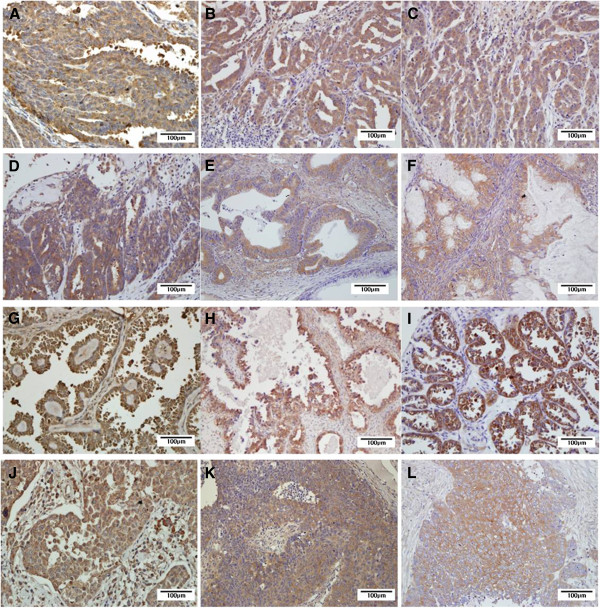
**OECs showed the expressions of TLR4, MyD88, and NF-κB.** (**A**) Serous type, TLR4, (**B**) serous type, MyD88, (**C**) serous type, NF-κB, (**D**) mucinous carcinoma, TLR4, (**E**) mucinous carcinoma, MyD88, (**F**) mucinous carcinoma, NF-κB, (**G**) clear cell type, TLR4, (**H**) clear cell type, MyD88, (**I**) clear cell type, NF-κB, (**J**) undifferentiated type, TLR4, (**K**) undifferentiated type, MyD88, (**L**) undifferentiated type, NF-κB. OECs, Ovarian epithelial cancers.

TLR4 protein was detected by immunohistochemistry and was localized in the cytoplasm and membrane of the tumor cells (Figure 1). The positive expression of TLR4 was observed in 57 cases (46.3%) of OECs. When analyzing the relationship between TLR4 expression and clinicopathological features, we found that the expression of TLR4 was not correlated with histologic grade, tumor size, mitotic count, tumor stage and tumor recurrence, but it was significantly correlated with histologic type. MyD88 was expressed in the cytoplasm of the tumor cells and the positive expression of MyD88 was observed in 45 cases (36.6%) of OECs, and it had a significant correlation with tumor stage, tumor recurrence and histologic type. NF-κB expression was recognized through cytoplasmic and/or nuclear staining of the tumor cells and was observed in 80 cases (60%) of OECs. No correlation between NF-κB expression and clinicopathological features was observed, except for histologic type. TLR4, MyD88, and NF-κB expressions were significantly higher in clear cell type of OECs than in the other histologic types of OECs (Table 2).

**Table 2:** Relationship between TLR4, MyD88 and NF-κB

		**TLR4**			**MyD88**			**NF-κB**		
		**Positive**	**Negative**	***P***	**Positive**	**Negative**	***P***	**Positive**	**Negative**	***P***
Histologic type	Serous	23	37	0.001	20	40	0.003	40	20	0.037
	Mucinous	6	19		5	20		15	10	
	Endometrioid	7	4		2	9		4	7	
	Clear cell	19	6		16	9		20	5	
	Undifferentiated	2	0		2	0		1	1	
Histologic grade	Grade 1	15	20	0.469	8	27	0.083	25	10	0.640
	Grade 2	32	30		24	38		39	23	
	Grade 3	10	16		13	13		16	10	
Tumor size	<10 cm	26	35	0.471	24	37	0.577	39	22	0.851
	≥10 cm	31	31		21	41		41	21	
Mitotic count	<10/10HPFs	25	25	0.569	16	34	0.660	35	15	0.541
	10 ≤ but <20/10HPFs	21	23		18	26		26	18	
	≥20/10HPFs	11	18		11	18		19	10	
FIGO stage	I/II	26	36	0.368	16	46	0.015	38	24	0.451
	III/IV	31	30		29	32		42	19	
Disease recurrence	No	27	35	0.589	16	46	0.015	40	22	0.526
	Yes	30	31		29	32		40	21	

TLR4 and MyD88 were co-expressed in 31 cases (25.2%) of OECs. TLR4, MyD88 and NF-κB were co-expressed in 26 cases (21.1%) of the OECs. The co-expression of TLR4/MyD88 or TLR4/MyD88/NF-κB in relation to clinicopathologic features is shown in Table 3. The co-expression of TLR4/MyD88 or TLR4/MyD88/NF-κB was significantly associated with tumor stage and histologic type. To study the association between TLR4, MyD88 and NF-κB, we performed the correlation analysis. The results showed that there was a significant correlation between the expression of TLR4 and MyD88 (r = 0.343, *P* = 0.000) (Table 4).

**Table 3:** Relationships between co-expression of TLR4/MyD88 signaling pathway proteins and clinicopathologic factors

		**TLR4/MyD88**			**TLR4/MyD88/NF-κB**		
		**Positive**	**Negative**	***P***	**Positive**	**Negative**	***P***
Histologic type	Serous	12	48	0.000	10	50	0.002
	Mucinous	2	23		1	24	
	Endometrioid	2	9		2	9	
	Clear cell	13	12		12	13	
	Undifferentiated	2	0		1	1	
Histologic grade	Grade 1	4	31	0.074	3	32	0.058
	Grade 2	20	42		18	44	
	Grade 3	7	19		5	21	
Tumor size	<10 cm	17	44	0.539	13	48	0.569
	≥10 cm	14	48		13	49	
Mitotic count	<10/10HPFs	10	40	0.415	9	41	0.707
	10 ≤ but < 20/10HPFs	14	30		11	33	
	≥20/10HPFs	7	22		6	23	
FIGO stage	I/II	10	52	0.023	8	54	0.028
	III/IV	21	40		18	43	
Disease recurrence	No	13	49	0.305	11	51	0.384
	Yes	18	43		15	46	

**Table 4:** Correlation among TLR4, MyD88 and NF-κB

		**TLR4**	**MyD88**	**NF-κB**
TLR4	Pearson Correlation	-	0.343	0.066
			0.000	0.469
	*P* value			
MyD88	Pearson Correlation	-	-	0.097
				0.287
	*P* value			

**Figure 2:**
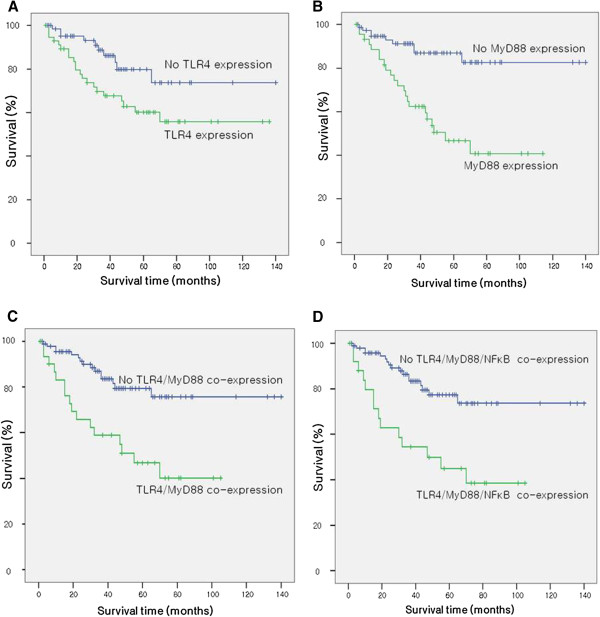
The Kaplan-Meier curves revealed the correlation between the expression of TLR4, MyD88, TLR4/MyD88 and TLR4/MyD88/NF-κB, and the overall survival (A-D).

We assessed the impact of expression of TLR4, MyD88 and NF-κB on patient survival. The mean survival of patients with expression of TLR4 in tumor tissues was 88.3 months, and it was 113.1 months in those with negative expression of TLR4, Kaplan-Meier analysis showed that there was a significant difference in the mean survival of patients with expression of TLR4 in tumor tissues and in those with negative expression of TLR4 (*P* = 0.030) (Figure 2A). Patients with positive MyD88 expression had a worse OS than those with negative MyD88 expression (65.2 months *versus* 121.1 months) (*P* = 0.000) (Figure 2B). Similar results were also found between co-expression of these proteins and OS (Table 5 and Figure 2C and 2D). In multivariate analysis, MyD88 was identified as an independent prognostic factor (Table 6).

**Table 5:** Univariate analysis of TLR4/MyD88 signaling pathway proteins on survival

**Protein**	**Expression**	**Means for survival time (months)**	**95% CI**		***P*value**
			**Lower**	**Upper**	
TLR4	Positive	88.3	72.5	104.1	0.030
	Negative	113.1	97.8	128.4	
MyD88	Positive	65.2	51.2	79.2	0.000
	Negative	121.1	109.4	132.8	
TLR4/MyD88	Positive	59.1	43.7	74.5	0.000
	Negative	113.9	101.9	125.9	
TLR4/MyD88/ NF-κB	Positive	56.5	39.5	73.5	0.000
	Negative	112.0	100.0	124.0	

**Table 6:** Multivariate analysis of TLR4/MyD88 signaling pathway proteins on survival

**Protein**	***P*value**	**Ratio of risk**	**95% CI**	
			**lower**	**upper**
TLR4	0.168	0.409	0.115	1.457
MyD88	0.004	0.128	0.032	0.513
TLR4/MyD88	0.438	2.580	0.235	28.300
TLR4/MyD88/NF-κB	0.551	0.553	0.079	3.883

## Discussion

TLRs are a family of PRRs that are best-known for their role in host defense against infection. There is increasing evidence that TLR signaling pathway is involved in tumorigenesis and chemoresistance. TLR-variants are known to be associated with cancer risk. Sequence variants of TLR4 are associated with prostate cancer risk and sequence variants of TLR4 and TLR10 are associated with nasopharyngeal cancer risk [12,13]. The risk of gastric carcinoma and some lymphoma subtypes is connected to polymorphism of TLRs [14].

TLRs are expressed on cells of the immune system but TLRs are also expressed on tumor cells. These expressions were observed in human tumor cells lines, such as colon, breast, and melanoma, as well as murine tumor cell lines, including colon, breast, prostate, lung and melanoma.

MyD88 plays a critical role in TLR signaling. TLRs generally signal through a MyD88-dependent manner, leading to a proinflammatory response. Signaling via MyD88 involves the early phase of NF-κB activation, which leads to the production of proinflammatory cytokines. Some experimental studies have identified the contribution of TLR4/MyD88 signaling to intestinal carcinogenesis. Wang *et al.*[15] demonstrated that TLR4 and MyD88 were expressed in tumor cells using immunohistochemistry on paraffin blocks of colorectal cancers and showed that TLR4/MyD88 expression was associated with liver metastasis and was an independent predictor of poor prognosis in patients with colorectal cancer. Their findings suggest that TLR4/MyD88 signaling promotes colorectal cancer progression by contributing to liver metastasis. In pancreatic ductal adenocarcinoma (PDAC), TLR4 was expressed in the tumor cells and was related to the survival of patients with PDAC [11].

In ovarian tumors, functional activity for TLR4 was demonstrated by stimulation of cell lines with specific ligands and subsequent activation and translocation of NF-κB and release of the proinflammatory cytokines interleukin-6 and CCL-2 [16]. Kelly *et al.* demonstrated for the first time the expression of TLR4 in OEC cells, the induction of tumor growth by TLR4 ligation in MyD88-positive OEC cells, and chemoresistance to paclitaxel mediated by the expression of MyD88. They also showed that patients whose tumors expressed MyD88 had a significantly worse progression-free interval compared with patient whose tumors did not express MyD88. In this study, we included a large number of cases of OECs and various histologic subtypes of OECs and tried to demonstrate the expression of TLR4/MyD88 signaling pathway proteins in tumor cells using an immunohistochemical method. We found that TLR4, MyD88 and N-FκB were frequently expressed in tumor cells of OECs and that expression of TLR4, MyD88, TLR4/MyD88 and TLR4/MyD88/NF-κB was associated with overall survival in patients with OECs. Particularly, MyD88 had an independent prognostic significance, and there was positive correlation between the expression of MyD88 and TLR4. Our findings suggest that TLR4/MyD88 signaling promotes OEC progression and that MyD88 plays a central role in OEC progression.

Our study also showed that the percentage of TLR4, MyD88 and NF-κB expression was significantly higher in the clear cell type of OEC than in the other types of OECs. Clear cell ovarian carcinoma has been known to show a poor prognosis, which is associated with the resistance to conventional platinum-based chemotherapy [17,18]. Several mechanisms have been proposed to be involved in drug resistance, including the lower proliferation of tumors, decreased drug accumulation, and increased DNA repair activity [18-20]. We mentioned the significance of TLR4/MyD88 signaling pathway in progression of OECs and suggest that the TLR4/MyD88 signaling pathway may be one of the mechanisms involved in drug resistance, and is especially associated with the poor prognosis of clear cell type of OECs.

Recent works have suggested a role for NF-κB in tumors of epithelial origin, including breast, colon, lung, and ovarian carcinomas [21,22]. Annunziata *et al.* reported a significant association of NF-κB p50 with poor overall survival in patients with OECs and suggested that the deregulation of NF-κB activity may influence outcome in patients who receive conventional chemotherapy for OECs. Our data showed that NF-κB p65 was frequently expressed in OECs, however, its expression was not associated with clinicopathologic factors of OECs, including overall survival. When NF-κB p65 was expressed in association with TLR4 and MyD88, it led to poor survival.

## Conclusions

Our study for the first time revealed that TLR4, MyD88, and NF-κB were expressed in the tumor cells of a large number of OECs using immunohistochemistry. Our data indicated that TLR4/MyD88 signaling pathway may contribute to progression of OECs and MyD88 expression is significantly associated with poor survival in patients with OECs. TLR4, MyD88 and NF-κB expression was more frequently observed in the clear cell type of OEC. These findings suggested the association of drug resistance with poor prognosis of clear cell type of OEC and the novel therapeutic possibility of targeting tumor cells.

## Abbreviations

TLR: Toll-like receptors; MyD88: Myeloid differentiation factor 88; NF-κB: nuclear factor- κB; OEC: Ovarian epithelial cancer; H&E: Hematoxylin and eosin; OS: overall survival; PDAC: pancreatic ductal adenocarcinoma; PRR: pattern recognition receptor.

## Competing interests

The authors declare that they have no competing interests.

## Authors’ contributions

KH and MS collected data, performed analysis, and drafted, revised and finalized the manuscript. KU conceived this study and participated in its design and coordination. DS, MS, DH, and JH revised and approved the contents of the manuscript. All authors read and approved the final manuscript.
